# Case report: Anesthetic management for removal of tumor thrombus in the inferior vena cava and pulmonary artery in renal cell carcinoma

**DOI:** 10.3389/fonc.2024.1372625

**Published:** 2024-03-18

**Authors:** Suli Chen, Liangyuan Lu, Xiangli Zheng, Yanjun Lin, Liming Bao, Bao Zhang, Zhanmin Yang

**Affiliations:** ^1^ Department of Anesthesiology, Aerospace Center Hospital, Peking University Aerospace School of Clinical Medicine, Beijing, China; ^2^ Department of Cardiac Surgery, Aerospace Center Hospital, Peking University Aerospace School of Clinical Medicine, Beijing, China; ^3^ Department of Urology, Aerospace Center Hospital, Peking University Aerospace School of Clinical Medicine, Beijing, China

**Keywords:** Renal cell carcinoma, tumor thrombus, inferior vena cava tumor thrombus, pulmonary artery thrombus, extracorporeal circulation, anesthesia management

## Abstract

Anesthetic management of patients with renal cell carcinoma with tumor thrombus in the inferior vena cava (IVC) is challenging. This paper reports the experience of anesthesia management in a patient with advanced renal cell carcinoma with thrombus accumulation in the IVC, right atrium, and pulmonary artery who underwent radical nephrectomy and tumor thrombus removal assisted by cardiopulmonary bypass. The emboli, measuring approximately 3 × 6 cm in the left inferior pulmonary artery and 4 × 13 cm in the right main pulmonary artery, were removed completely. During incision of the IVC under systemic heparinization, significant blood loss occurred in the surgical field. The surgery took 724 min, and cardiopulmonary bypass took 396 min. Intraoperative blood loss was 22,000 ml. The patient was extubated 39 hours after surgery and stayed in intensive care unit for 3 days. At 1 year follow-up, the patient was in good health and leading a normal life.

## Introduction

Renal cell carcinoma (RCC) is the second most common type of urological cancer worldwide. Carcinoma cells can accumulate in the renal veins, resulting in the formation of a renal vein tumor thrombus. Continuous growth of the tumor thrombus may lead to its extension into the inferior vena cava (IVC) and even reach the right atrium. The incidence of renal cell carcinoma combined with venous tumor thrombus is 4%–10% in all renal cell carcinoma cases (1). Reports of tumor thrombus accumulating simultaneously in the right atrium and pulmonary artery are rare.

In the past, patients with renal cell carcinoma complicated by venous thrombosis were considered to be in an advanced stage, and the opportunity for surgical treatment was limited. Moreover, surgical procedures are difficult, high-risk, and challenging to perform. In recent years, with continuous improvements in surgical techniques, radical nephrectomy and thrombectomy have become important treatment methods for patients with renal cancer and venous thrombus (2, 3). However, once the thrombus dislodges, the risk of death is extremely high, especially for Mayo Clinic stage IV patients. To improve anesthesia management, reduce complications, and improve survival rate of such patients, we herein report a case of anesthesia management during removal of tumor thrombus in the IVC and pulmonary artery in renal cell carcinoma. The patient provided written consent to publish this case. This study was approved by the Institutional Research Ethics Committee of the Aerospace Center Hospital.

## Case presentation

### Patient information

**Figure 1:**
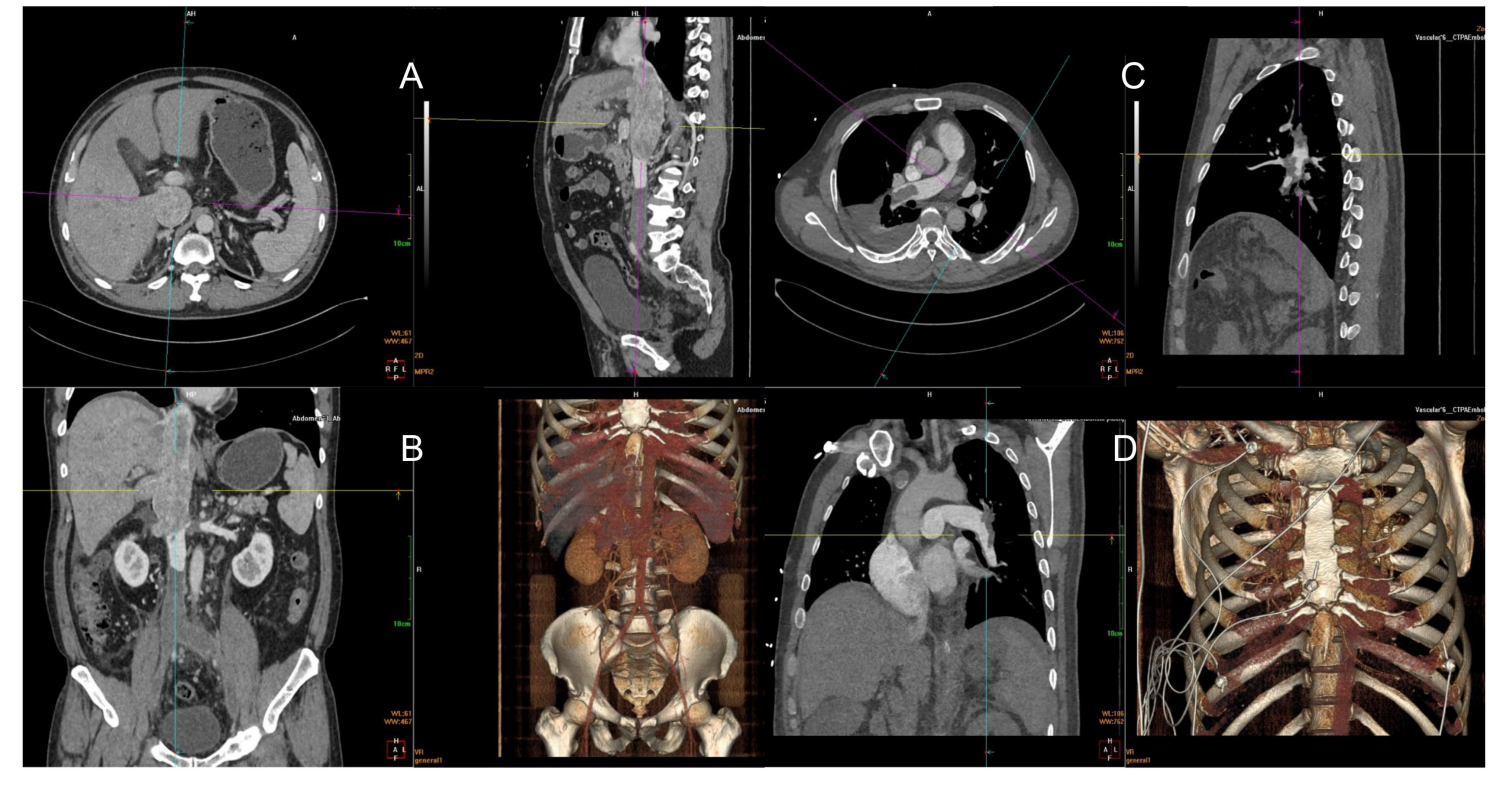
Filling defects from the beginning of the right renal vein and bilateral iliac veins to the inferior vena cava atrium, diffuse and filled with low-density shadow **(A, B)**. Filling defect in the left lower pulmonary artery **(C)**. Multiple filling defects can be seen in the left lower lobe pulmonary artery trunk and its branches, right pulmonary artery trunk and its branches, and left upper lobe pulmonary artery branches **(D)**.

A 50-year-old man (height, 181 cm; weight, 94 kg) was admitted to the hospital for “lower limb vein thrombosis” and “filter placement.” He was diagnosed with “right renal cell carcinoma and IVC tumor thrombus” 8 days before the surgery. Enhanced computed tomography (CT) revealed thrombus spreading from the renal vein into the vena cava, right heart atrium, and pulmonary artery. Bilateral common iliac vein, external iliac vein, and femoral vein were secondary apposition blood clot formation. (**Figure 1A, B**). Weak blood flow imaging was detected at the embolization of IVC. Three-dimensional pulmonary artery CT suggested the presence of a left lower pulmonary artery embolism. The patient had complained of poor appetite, abdominal distension, and vomiting, accompanied by wheezing and progressive exacerbation. Physical examination revealed the absence of respiratory sounds in the right lower lung. Urgent three-dimensional pulmonary artery CT revealed multiple pulmonary embolisms, including multiple filling defects in the left lower lobe pulmonary artery trunk and its branches, left upper lobe pulmonary artery and its branches, and right pulmonary artery trunk and its branches (**Figure 1C, D**).

Preoperative MRI examination wasn’t performed to assess the risk of IVC wall tumor infiltration because the patient’s condition deteriorated rapidly after coming to our hospital, and emergency operation had to be performed. The ASA classification of patient was level IV. 20 units packed RBCs, 2000ml fresh frozen plasma (FFP) and 3 units platelets were prepared before surgery.

### Preoperative diagnosis

Right kidney malignant tumor, IVC tumor thrombus, pulmonary artery embolism, lower limb vein embolism, bilateral common iliac vein embolism, bilateral external iliac vein embolism, and right pleural effusion.

### Anesthesia management

After the patient entered the operating room, two 16G peripheral intravenous catheters were inserted to establish venous access before anesthesia induction. Monitoring devices including invasive artery blood pressure (IBP), electrocardiography, pulse oxygen saturation, and bispectral index, body temperature (rectal and nasal temperature), and FloTrac-Vigileo were attached. For anesthesia induction the following medications were administered sequentially: 0.5mg of penehyclidine, 20mg of etomidate, 30ug of sufentanil, and 20mg of benzenesulfonate cisatracurium. Tracheal intubation (7.5# enhanced endotracheal tube) was successfully completed using a visual laryngoscope. The tidal volume was maintained at 5–7 ml/kg, the respiratory rate was 12–14 times/minute, the inhalation:expiration ratio was 1:2 and PEEP was 0cmH_2_O in the volume-controlled ventilation mode. P_ET_CO_2_ was maintained between 35 and 45 mmHg. To maintain anesthesia, 1%–1.5% sevoflurane was continuously administered through inhalation, propofol was pumped continuously at 15–20ml/h, and sufentanil, midazolam, and benzenesulfonate cisatracurium were injected intermittently. A three-lumen central venous catheter (7F) was placed through the right internal jugular vein to monitor central venous pressure (CVP) and administer vasoactive drugs. The internal environment and acid-base balance were adjusted according to blood gas analysis results.

### Intra-operative anesthesia

**Figure 2:**
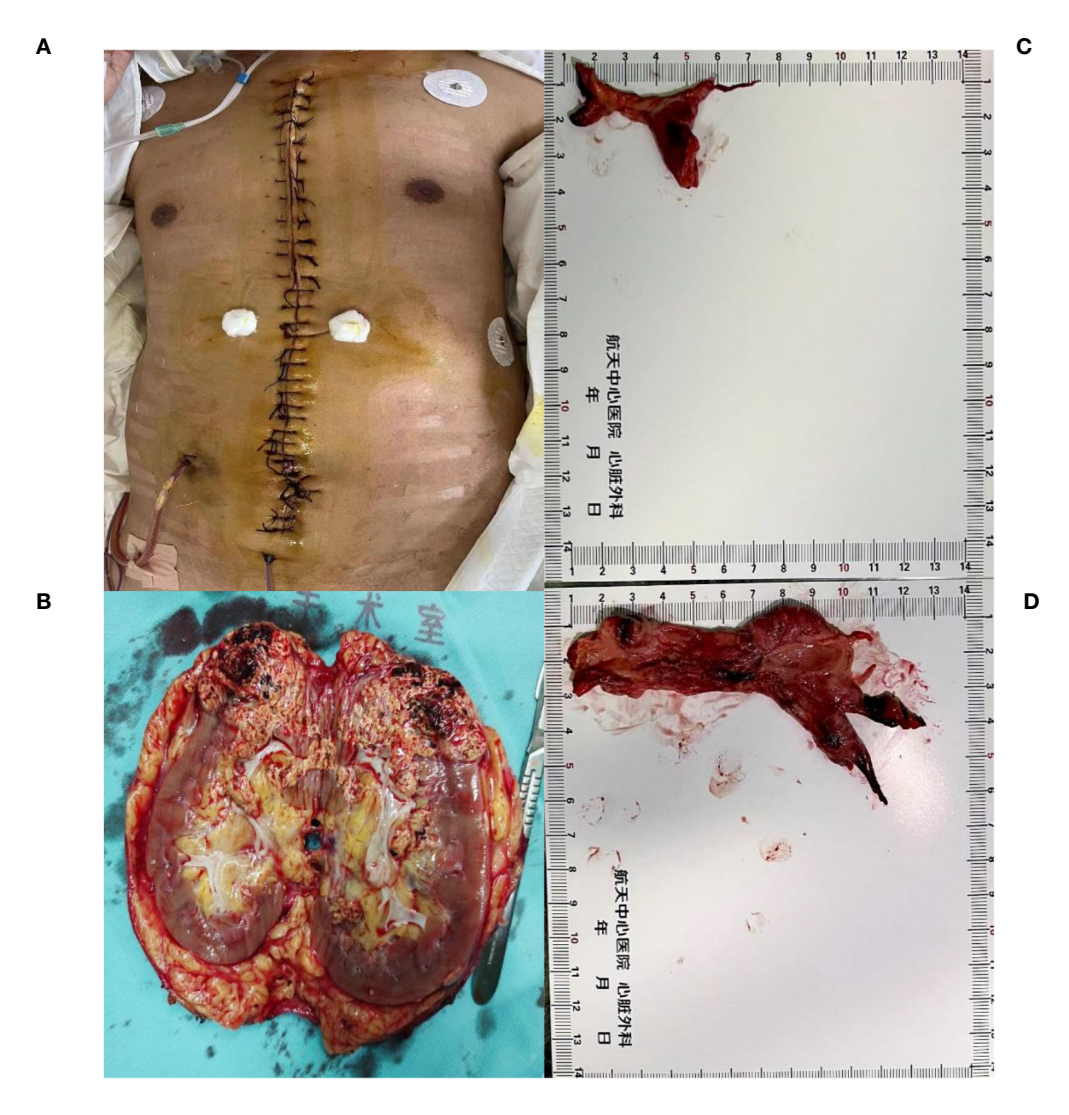
The removed kidney is 15*8*7 cm in size, with visible renal vein tumor thrombus **(A, B)**.Tumor thrombus at the opening of the left inferior pulmonary artery and tumor thrombus at the main trunk of the right pulmonary artery, with a size of about 3*6cm **(C)** and 4*13 cm **(D)**.

After successful induction of anesthesia, we performed a median sternal incision (**Figure 2A**). On opening the pericardium, we observed that the heart had become spherical due to significantly increased pressure in the right atrium, right ventricle, and pulmonary artery. We first performed aortic and axillary vein catheterization to try to establish extracorporeal circulation because of the right atrial thrombus occupancy. But the flow rate of cardiopulmonary bypass (2.8L/min) cannot be satisfied. The surgeon decided to open the right atrium to explore the thrombus and attempt to remove the IVC thrombus before intubating the IVC. However, no tumor thrombus was found in the right atrial segment of the IVC during exploration, and IVC catheterization was performed. The extracorporeal circulation flow rate reached 4.9L/min, the ascending aorta was blocked, and 2000ml of cardiac arrest fluid HTK was administered. After cardiac arrest, no tumor thrombus was found in the right atrium or right ventricle. A patent foramen ovale with a diameter of approximately 5 mm was observed. The main pulmonary artery was cut longitudinally to the main trunk of the left and right pulmonary arteries, and the emboli were blocked at the opening of the left inferior pulmonary artery and main trunk of the right pulmonary artery. The emboli, measuring approximately 3×6 cm in the left inferior pulmonary artery and 4×13 cm in the right main pulmonary artery, were removed completely. The distal end of the emboli had a fork (**Figure 2C, D**). This process proceeded smoothly. Subsequently, the temperature increased, and the heart resumed beating (The aortic cross-clamp time was 84 minutes). Low dose of dobutamine, norepinephrine, and milrinone were used to maintain the hemodynamic stability.

To prevent the thrombus from dislodging, we kept the IVC unopened and continued cardiopulmonary bypass. The urologists chose the median abdominal incision approach for right nephrectomy (**Figure 2A, B**) and IVC thrombectomy. During incision of the IVC under systemic heparinization, significant blood loss occurred in the surgical field. The patient’s hemodynamics fluctuated severely, with average IBP as low as 40 mmHg. At this time, owing to blood loss in the surgical field, the return flow of the vena cava during extracorporeal circulation decreased sharply. Supplementary crystal solutions, colloid solutions, and red blood cell (RBC) transfusions were provided. A blood recovery machine was used to collect and wash the blood lost in the surgical field before reinfusing it into the patient’s body. A higher doses of vasoconstrictor and positive inotropic drugs were administered simultaneously and maintain IBP fluctuations within the range of 80(70-100)/45(40-60) mmHg and a heart rate of 100–135 beats per minute. The CVP fluctuated between 1 and 5 cmH_2_O.

In order to reduce the potential damage caused by organ hypoxia, we maintained the body temperature between 34 and 35 °C. After completing the main procedure, protamine was administrated to neutralize heparin (Guided by activated coagulation time). Then, blood products were given to correct the clotting dysfunction. The surgery took 724 min, and cardiopulmonary bypass took 396 min. The total output was 24700 ml, including 22000 ml of bleeding and 2700 ml of urine. The total intake included 9500 ml of crystals, 4000 ml of colloids(succinyl gelatin), 32 U of suspended RBCs(including 18 U for extracorporeal circulation infusion), 2600 ml of FFP; 4 U of platelets, 6876 ml of washed RBCs, 6g of fibrinogen, and 800IU of prothrombin complex. A total of 710 ml 0.5% NaHCO_3_ was infused to correct acid-base balance based on the results of blood gas analysis.

### Pathological report

Clear cell renal cell carcinoma of the right kidney (T4N_X_M_X_). The thrombi in the left pulmonary artery, right pulmonary artery, and IVC tumor showed features of clear cell renal cell carcinoma with extensive necrosis.

### Post-operation management

The postoperative hemodynamics of the patient were stable and the recovery was smooth. A total of about 300ml of fluid was drained from the locations of intro-abdominal, chest drainage. Only about 700ml of FFP was transfused to correct coagulation disorders after surgery. The patient recovered successfully. Abnormal serum creatinine lasted for 2 days (up to 214.4umol/L) and urine volume was normal. The patient was extubated 39 hours after surgery and was hospitalized in the intensive care unit for 3 days without other serious complications. Rivaroxaban was routinely administrated to prevent thrombosis. 5mg of Axitinib and 240mg of Treprizumab were used for antitumor treatment.

### Follow-up

Two months after the surgery, the patient returned to the hospital for routine examination. Enhanced CT showed that there were still a few thrombus in the inferior vena cava, bilateral iliac vein, and the lower lobes of both lungs, but there was no significant change compared with the postoperative results. One year later, the patient returned to the hospital for examination, and there was no obvious change in the condition compared with before.

## Discussion

In this case, a patient with renal cell carcinoma developed venous thrombosis of the lower limb first, followed by the rapid development of symptoms of obstruction of the right heart system and pulmonary embolism, such as anorexia, abdominal distention, vomiting, and progressive dyspnea. The only way to save the patient’s life was to remove the tumor thrombus as soon as possible to remove the obstruction and restore the patency of the lumen. Previous studies have reported that the 5-year survival rate of renal cancer patients without lymph node or distant metastasis after radical nephrectomy and thrombectomy is approximately 50% (4, 5). In the last decade, the development of robotics and improvement of surgical techniques provided new methods for treatment of level I to IV IVC thrombus (6–8). But the case reported here is extremely rare.

Schmidt first reported tumor-induced pulmonary embolism in 1987. The incidence of tumor-induced pulmonary embolism in patients with substantive and malignant tumors is 2.4%–26% (9), and the perioperative mortality rate is as high as 9.7%–22.2%. Pulmonary artery embolism is a fatal perioperative complication in patients with renal cancer, with only a few reports of successful thrombectomy under extracorporeal circulation (10). This patient experienced a recurrent peripheral vascular embolism before surgery, resulting in obstruction of the left and right pulmonary artery trunks, which is extremely rare. Therefore, a multidisciplinary collaborative thoracoabdominal surgery was performed. Preoperative imaging showed that the IVC was almost entirely filled by the tumor thrombus, which also appeared to extend into the right atrium. However, no tumor thrombus was found in the right atrium or proximal end of the IVC during surgical exploration. We considered that increasing venous pressure caused by the almost completely blocked inferior vena cava thrombosis forced the embolus to move to the pulmonary artery. Therefore, the patient experienced rapid deterioration after admission, especially increased dyspnea. Intraoperative exploration revealed that the right main pulmonary artery and left inferior pulmonary artery were blocked by the tumor thrombus.

The patient had multiple embolisms in the left lower lobe artery, left upper lobe artery, and right pulmonary artery trunk. While severe pulmonary artery occlusion due to embolism typically leads to acute right heart failure in most patients, this patient did not show symptoms of acute right heart failure. We believe that this may be due to the reopening of the foramen ovale following a sharp increase in right heart pressure, which alleviated some of the pressure on the right heart, as confirmed during surgical exploration.

To avoid the risk of pulmonary artery re-embolism due to tumor thrombus detachment during surgery, the surgeon chose not to open the IVC after removal of the pulmonary artery tumor thrombus. Opening the IVC after completing radical nephrectomy and thrombectomy of the IVC under extracorporeal circulation avoids the risk of recurrent pulmonary embolism during surgery.

In the present case, the tumor was large, extending from the femoral vein to the beginning of the IVC atrium, and the tumor thrombus heavily adhered to the IVC. During the removal of the thrombus, the IVC was torn and the amount of bleeding reached 22000 ml. For this type of massive bleeding, in addition to actively using vasoactive drugs to maintain the circulation, quick and extensive transfusions and replenishment of fluids is important. Significant blood loss in the surgical field can result in insufficient extracorporeal circulation recovery; therefore, timely RBC transfusion and crystals colloids supplementation are required to maintain fluid level stability. Blood loss in the surgical field can also be treated using a blood recovery device to replenish lost blood to the circulation through peripheral veins. Previous studies have indicated that intraoperative blood salvage was not associated with long-term tumor recurrence (11, 12). In this patient, given his life-threatening condition due to fatal massive bleeding, we believe that the use of recovered and reused surgical field blood to replenish blood volume was justified. Moreover, we did not observe any signs of tumor metastasis to other organs during long-term follow-up of the patient.

Maintaining a normal body temperature is essential for the normal physiological functioning of organs and the maintenance of coagulation function. However, in this case, when the thrombus was removed by cutting the IVC under cardiopulmonary bypass, the amount of bleeding caused by surgical factors was large. Hypoperfusion and hypothermia inevitably occur with the use of large amounts of vasoconstrictor drugs and rapid infusion of low-temperature blood components. Numerous studies have demonstrated that maintaining the body in a mildly hypothermic state can have a protective effect on damaged brain tissue (13, 14). Low body temperature can also alter platelet aggregation and reduce enzyme activity in the coagulation cascade reactions (15, 16). These changes inevitably increase the risk of perioperative bleeding and blood transfusion. Research has shown that keeping patients warm during the perioperative period can prevent blood loss and transfusion needs caused by unexpected hypothermia during the perioperative period (17). In this case, during thrombectomy of the IVC assisted by cardiopulmonary bypass, we maintained the patient’s temperature in the range of mild hypothermia, which may have reduced the consumption of coagulation factors. After the completion of the main surgical steps, the patient’s body temperature was gradually returned to normal. Subsequent supplementation with coagulation factors ensured that the patient did not experience severe postoperative blood loss. Based on the patient’s postoperative recovery, we believe that these intraoperative treatments were of great significance.

Maintaining clotting function in patients undergoing such surgery is challenging. Previous reported the survival rate of patients with massive blood volume supplementation and reabnormal coagulation could be improved by using high FFP: RBC ratio and high platelet: RBC ratio (18). Although, RBC transfusion is the most important protective measure to maintain the oxygen supply of organs during massive blood loss, maintaining clotting function is more challenging. After protamine neutralization of heparin, FFP and platelets, which account for about 30% of blood volume, were transfused firstly. Then, additional coagulant factors including fibrinogen, and prothrombin complex were administrated under the guidance of blood routine, coagulation function and thromboelastography. The ratio of total RBCs, fresh frozen plasma and platelets transfused was close to 1:1:1.

Although a fluke success was lucky, excessive blood loss can be fatal to a similar operation. Previous studies reported that use of cardiopulmonary bypass with deep hypothermic circulatory arrest, with the advantage of selective aortic arch perfusion, allowed for a safe, precise, complete extirpation of intracaval and intracardiac tumor mass, and less risks of blood loss (19, 20). The Late outcomes after radical surgical treatment in patients with RCC and tumor thrombus reaching up in the right atrium can be 51% in the 5-year cancer-related survival rate. Therefore, this clinical condition indisputably posed a serious challenge for a proper surgical strategy.

In addition, regarding postoperative analgesia, previous studies have reported that ultrasound-guided transabdominal plane block can reduce morphine consumption and physical pain, thereby reducing the incidence of chronic pain and improving postoperative pain management (21, 22). Present patient received intravenous opioid analgesia after surgery and responded well. However, we believe that multimodal analgesia may be a good option in patients with similar surgery.

## Conclusion

For Mayo Clinic stage IV patients with advanced renal cell carcinoma with tumor thrombus in the IVC and pulmonary artery, extracorporeal circulation and multidisciplinary collaboration are reasonable choices. However, surgery poses more complex challenges in such patients.

## Data availability statement

The raw data supporting the conclusions of this article will be made available by the authors, without undue reservation.

## Ethics statement

The studies involving humans were approved by Institutional Research Ethic Committee of the Aerospace Center Hospital. The studies were conducted in accordance with the local legislation and institutional requirements. The participants provided their written informed consent to participate in this study. Written informed consent was obtained from the individual(s) for the publication of any potentially identifiable images or data included in this article. Written informed consent was obtained from the participant/patient(s) for the publication of this case report.

## Author contributions

SC: Writing – original draft, Investigation, Data curation. LL: Writing – review & editing, Writing – original draft, Supervision. XZ: Writing – review & editing, Investigation, Data curation. YL: Writing – review & editing, Resources, Data curation, Conceptualization. LB: Writing – review & editing, Project administration, Methodology. BZ: Writing – review & editing, Project administration, Methodology. ZY: Writing – review & editing.
